# Adaptations to the stressful combination of serpentine soils and Mediterranean climate drive plant functional groups and trait richness

**DOI:** 10.3389/fpls.2023.1040839

**Published:** 2023-03-13

**Authors:** Noelia Hidalgo-Triana, Andrés V. Pérez-Latorre, Aristide Cossi Adomou, Michael Rudner, James H. Thorne

**Affiliations:** ^1^ Botany and Plant Physiology Department, University of Malaga, Málaga, Spain; ^2^ Department of Plant Biology, University of Abomey-Calavi, Abomey-Calavi, Benin; ^3^ Faculty of Environmental Engineering, Weihenstephan-Triesdorf University of Applied Sciences, Weidenbach, Germany; ^4^ Department of Environmental Science and Policy, University of California, Davis, Davis, CA, United States

**Keywords:** ultramafic vegetation, functional traits, functional groups, adaptations, Mediterranean climate, serpentinophytes, generalists, drought avoidance

## Abstract

**Introduction:**

Plant functional traits (FTs) are important for understanding plant ecological strategies (e.g., drought avoidance), especially in the nutrient-poor soils of serpentine ecosystems. In the Mediterranean areas, such ecosystems are characterized by climatic factors (e.g., summer drought) that exert a filtering effect.

**Material and Methods:**

In our study, we analyzed 24 species with varying serpentine affinity, from strictly serpentine plants to generalist plants, from two ultramafic shrublands in southern Spain, considering four FTs: plant height (H), leaf area (LA), specific leaf area (SLA), and stem specific density (SSD). Additionally, we also identified the species’ dominant strategies to avoid drought and those strategies’ relationship to serpentine affinity. We used principal component analysis to identify combinations of FTs, and cluster analysis to define Functional Groups (FGs).

**Results and Discussion:**

We defined eight FGs, which suggests that such Mediterranean serpentine shrublands are composed of species with wide-ranging of FTs. Indicator traits explained 67–72% of the variability based on four strategies: (1) lower H than in other Mediterranean ecosystems; (2) middling SSD; (3) low LA; and (4) low SLA due to thick and/or dense leaves, which contribute to long leaf survival, nutrient retention, and protection from desiccation and herbivory. Generalist plants had higher SLA than obligate serpentine plants, whereas the obligate serpentine plants showed more drought avoidance mechanisms than the generalists. Although most plant species inhabiting Mediterranean serpentine ecosystems have shown similar ecological adaptations in response to the Mediterranean environment, our results suggest that serpentine obligate plant species could present greater resilience to climate change. Given greater number and more pronounced drought avoidance mechanisms in these species compared with generalists, and the high number of FGs identified, the serpentine plants have shown adaptation to severe drought.

## 1 Introduction

Plant communities in environmentally harsh serpentine soils may demonstrate unique responses to environmental change, *via* the mediating role of their functional traits (FTs; Adamidis et al., 2013). Serpentine soils, deriving from ultramafic rock and found along continental margins and orogenic belts worldwide, contain high levels of magnesium and high concentrations of heavy metals, particularly chromium and nickel, as well as low levels of calcium, nitrogen, potassium, and phosphorus (Kruckberg, 1984; Adamidis et al., 2013; Anacker, 2014). Although some plant species have developed various affinities for growing in serpentine soils, most cannot tolerate those conditions (Pérez Latorre et al., 2013; Pérez-Latorre et al., 2018). The plants that adapt are often endemics (i.e., obligate serpentinophytes), do not occur in non-serpentine soils, and form distinctive assemblages, often referred to as ultramafic vegetation (Sánchez-Mata and Rodríguez-Rojo, 2016). While ultramafic vegetation is distributed globally, the Western Mediterranean Basin contains some of the world’s most species-rich serpentine flora (Selvi, 2007).

FTs are morpho-physio-phenological traits that are described as key components of ecosystem properties (Cornelissen et al., 2003; Violle et al., 2007; Damschen et al., 2012; Breitschwerdt et al., 2018; Green et al., 2022). Analyzing FTs is increasingly used to elucidate the role of particular traits in the functioning of plant communities (Grime, 2006; Lavorel et al., 2011). Although information about plant FTs is available for a large portion of the global pool of plant species including the Plant Trait Database (TRY- Kattge et al., 2011) and others (i.e. Wright et al., 2004; Breitschwerdt et al., 2018), such global databases do not include complete information about FTs for serpentine plants, which should show adaptations to harsh soil environments (Kazakou et al., 2008; Harrison et al., 2017). Instead, only general traits have been identified for worldwide serpentine-endemic flora, including reduced specific leaf area (SLA), sclerophyllous leaves, and shorter heights compared with non-serpentine plants (Harrison and Rajakaruna, 2011; Hidalgo-Triana and Pérez Latorre, 2019).

A commonly used technique for quantifying functional diversity in ecosystems consists of clustering species with shared taxonomic, physiological, and morphological traits into Functional Groups (FGs) whose species share FTs (Petchey and Gaston, 2002; Zanzottera et al., 2020). Trait similarity among species in plant FGs, reflects their similar adaptations and responses to environmental conditions (Violle et al., 2007; Breitschwerdt et al., 2018). In general, functional diversity is low in regions of strong abiotic stress but higher in regions where competitive interactions are stronger (Cornwell and Ackerly, 2009; Mason and de Bello, 2013). However, results for serpentine areas are relatively mixed. Hobbie et al. (2012) have shown that dry, and infertile serpentine soils, are dominated by a mixture of FGs, while Fernandez-Going et al. (2012) found low functional diversity in a serpentine grassland in California. By contrast, Hidalgo-Triana et al. (2018) found a high functional diversity in serpentine chaparral.

There are no studies on the FGs of serpentine vegetation in Mediterranean ecosystems, despite the fact that the climatic factor in Mediterranean serpentine ecosystems exerts a filtering effect. Therein, dry summer conditions may favor species with traits that allow them to use nutrient and water resources more conservatively (Adamidis et al., 2014). Harrison et al. (2017) have concluded that grassland plant communities in infertile serpentine soils are less sensitive to changes in the climatic water balance than communities in more fertile soils. However, it is important to consider whether species occurring only in a narrow set of environmental conditions, such as those found in serpentine flora in the Mediterranean Basin, may be particularly susceptible to the effects of a warming climate (Damschen et al., 2010; IPCC, 2018).

Current global circulation models (GCMs, e.g., Eyring et al., 2016) predict more frequent, more severe extreme climatic events such as droughts (Mittal et al., 2014). In serpentine flora, species-specific structural characteristics such as conservative mechanisms linked to the adaptation of water stress conditions are frequent (Sardans and Peñuelas, 2013). For example, seasonal dimorphism in semideciduous species (Puglielli and Varone, 2018) consists of the reduction of their green structures to brachyblasts during summer (Orshan, 1964; Orshan, 1989). At the morphological level of foliage, Mediterranean plant species may improve their capacity for drought avoidance with increased foliar sclerophylly (i.e., the development of thick cuticles and increased leaf mass area), a high density of foliar trichomes, high plasticity in foliar morphology, decreased size (Sardans and Peñuelas, 2013), and photosynthetic stems/stem-like leaves (Travlos and Chachalis, 2008). Because Mediterranean vegetation is generally considered to be well adapted to drought conditions, the combination of serpentine soils and the Mediterranean climate that occurs in Mediterranean serpentine vegetation is expected to present more drought-tolerant species (Garssen et al., 2014). Those serpentine ecosystems can be regarded as promising environments for studies on plant FTs due to the resilience that FTs may confer under climate change. In addition, those ecosystems contain plant species exhibiting a range of serpentine affinities (Pérez Latorre et al., 2013; Pérez-Latorre et al., 2018), including edaphic endemics that grow solely in serpentine soil and generalist species that may occur there but are more common in other soils (Damschen et al., 2012).

In our study, we sought to identify traits that allow serpentine species a relative advantage under worsening conditions due to climate change. To that end, we measured FTs and FGs associated with obligate serpentine species and with more generalist plant species inhabiting the same serpentine soil. Overall, we sought to determine whether the existence of drought tolerance is conditioned by the range of serpentine affinity and driven by the xeric conditions of serpentine soils.

We had three objectives:

1. To characterize the FTs of south Iberian serpentine plants distributed across two Mediterranean serpentine shrublands and to develop a new plant trait database containing records of serpentine Mediterranean shrublands;
2. To create a classification of FGs for the two south Iberian serpentine shrublands and characterize patterns in community-weighted mean trait values in the two communities, and;
3. The new database was used to assess the relationships among serpentine affinities, ecological characteristics of the shrublands, and drought avoidance mechanisms.

## 2 Materials and methods

### 2.1 Study site

The study was conducted on the ultramafic outcrop of Sierra Bermeja (municipality of Estepona, Malaga, Andalusia, Spain), where vegetation is mainly dominated by *Pinus pinaster* forests (Mota et al., 2017). However, disturbance processes have promoted the dominance of two xerophytic shrubland types in those serpentine ecosystems (Mota et al., 2017). Those types of shrublands occupy clearings in *P. pinaster* forests but differ in their elevations and floristic composition. They are also where most edaphic endemic plant species occur, along with other species that are tolerant of but not restricted to serpentine.

We selected areas occupied by those two existing types of shrubland (see **Supplementary Material 1 Figure S1**). The first, at 590 m, is considered a thermomediterranean *Halimium atriplicifolium* shrubland (HS) developed on entisols, while the second, at 1270 m, is a mesomediterranean *Cistus populifolius* shrubland (CS), also developed on entisols.

We surveyed one vegetation plot measuring 20 m x 20 m (400 m^2^) in each type of shrubland. To gather floristic composition data, plant inventories were made following Braun-Blanquet (1979), and the vegetation was classified following the European classification of natural habitats (European Commission, 2013) belonging to code 5330 (Thermo-Mediterranean and pre-desert scrub). Climatic data were obtained from Gómez-Zotano et al. (2016) and soil textural classes were established following the USDA: Rowell D (2014). The two studied sites are characterized by different elevation and thus different floristic composition.

### 2.2 Selection of species and traits

From 2013 to 2018, woody and perennial herbaceous plant species exhibiting morphological–functional adaptations to the Mediterranean dry season were selected (Valencia et al., 2016). The range of species selected was designed to represent a continuous range of leaf traits based on prior ecological and floristic knowledge (Pérez Latorre et al., 2013; Pérez-Latorre et al., 2018; see **Supplementary Material 2 Table S1**). We selected 24 species, subspecies, and varieties (hereafter “species”): 18 in the HS shrubland and 18 in the CS shrubland, belonging to 14 taxonomic families and 21 genera (see **Supplementary Material 2 Figure S1**). Taxonomic nomenclature follows Blanca et al. (2011) combined with taxonomic synonyms according to POWO (2019). Each species’ affinity to serpentine soils in the region can be considered on a continuum, ranging from obligate to preferential to generalist (Pérez Latorre et al., 2013; Pérez-Latorre et al., 2018). Categories of species’ affinity for peridotite and serpentine soils were identified using three categories: obligate (O), which are considered to be strictly serpentine plants; preferential (P), which are serpentine plants with populations located “primarily” in serpentine soils; and broad-spectrum (BS), which are generalist species that can survive in a wide variety of soils (Blanca et al., 2011; Pérez Latorre et al., 2013; Pérez-Latorre et al., 2018; see **Supplementary Material 2 Table S1**). According to Raunkiaer (1934), the taxa in our study were primarily comprised in the biological types of chamaephytes with some dwarf phanerophytes (nanophanerophytes). Growth height is considered to be more or less equal to 0.6 m for phanerophytes and less than 0.6 m for chamaephytes. Voucher specimens for each species were accessioned to the MGC Herbarium (University of Malaga, Spain).

For each plant species, we collected FT data following the standardized criteria stipulating that ten replicates are needed for leaf traits and 25 for height (Cornelissen et al., 2003; Pérez-Harguindeguy et al., 2013). FTs measured included (see **Supplementary Material 3 Table S1**): plant height (H), traits in relation to photosynthetic organs (leaf area (LA) and specific leaf area (SLA)), and stem specific density (SSD). H (in cm) is the shortest distance between the upper boundary of the main photosynthetic tissues (excluding inflorescences) on a plant and the ground level. For H, we sampled 25 healthy plants. SLA (in mm^2^/mg) is the one-sided area of a fresh leaf (LA), divided by its oven-dry mass. We collected ten leaves from at least ten individuals of each studied species in each shrubland. Leaves were transported to the laboratory in plastic bags and stored at low temperatures (2–6 °C) for less than 24 h prior to measurement and leaf dry mass was determined after oven drying at 60°C for at least 72 h. Leaf area (LA in mm^2^) measurements of each individual leaf were estimated using ImageJ software. SSD (in mg/mm³) is the oven-dry mass (at 70 °C for 72h) of a section of the main stem of a plant divided by the volume of the same section, when still fresh (Pérez-Harguindeguy et al., 2013).

Because drought is a major limitation to plant productivity in Mediterranean ecosystems (Adamidis et al., 2014), we also measured the presence and absence of drought avoidance leaf traits in the field (i.e., July 2017), using the same replicates used for the FT study. Drought avoidance leaf traits measured were: seasonal dimorphism with leaf rolling during stress, leaves containing more trichomes (Sardans and Peñuelas, 2013), plants with photosynthetic stems or green leafy shoots (stem-like leaf; Travlos and Chachalis, 2008). See **Supplementary Material 2 Table S1**.

### 2.3 Statistical approach

To address objective 1, for all measures of each species, we calculated the means, standard deviation (SD) and coefficient of variation (CV, defined as SD/Mean), of their defining traits for two levels of analysis: for each species (i.e., full data set) and for the species found in the two types of shrublands (i.e., CS and HS).

We used the Shapiro–Wilk test to confirm non-normality assumptions (P <0.05). A non-parametric one-way Analysis of Variance (ANOVA) was used to explore differences in traits among the two shrublands (HS and CS).

To classify the species (objective 2), we used the major axes of variation in their FTs. Prior to analysis, the variables were normalized by subtracting the mean and dividing by the standard deviation. Principal component analysis (PCA) was used for each component (i.e., normalized var-covar)) using H, LA, SLA, SSD in each shrubland studied. Agglomerative clustering (dendrogram) based on Euclidean distances and Ward Algorithm was performed, using all the FTs to define FGs and those of each shrubland. The dendrogram cut level was determined visually with the goal of maintaining a minimum Euclidian distance, maximizing the variance between clusters and aligning with field observations of FTs, in order to make the resulting groups interpretable. For each FG obtained, life form was included considering Raunkiaer (1934) categories. We used the Strategies Richness index (Sri) to measure the richness of strategies characterizing each type of shrubland calculated as the ratio between the number of FGs and the total number of species in the community (Pérez Latorre et al., 2007).

To address objective 3, we performed a chi-square (χ^2^) test to assess the relationships among serpentine affinities, ecological characteristics of the shrublands, and drought avoidance mechanisms.

Cluster analysis and PCA were performed with the free software PAST (version 2.17, Hammer et al., 2001). For the rest of the analyses, we used SPSS (version 18.0, SPSS Inc., USA).

## 3 Results

### 3.1 Characterization and variation of species FTs

Plant height (H) showed a mean value of 40.73 cm for all species (**Table 1**) and ranged from 10.75 cm (i.e., *Carex distachya* in CS) to 126.64 cm (*Erica scoparia* in HS). SLA showed a mean value of 8.43 mm²·mg-¹ (**Table 1**) and ranged from 1.73 mm²·mg-¹ in *Halimium atriplicifolium* (HS) to 26.38 mm²·mg-¹ in the *Genista triacanthos* (CS) (see **Supplementary Material 2 Table S1**). LA resulted in a mean value of 123.27 mm^2^ and ranged from 4.75 mm^2^ in *Sanguisorba verrucosa* in CS to 1555.58 mm^2^ in *Cistus salvifolius* in CS (see **Supplementary Material 2 Table S1**). SSD reached a mean value of 0.67 mg/mm³ (**Table 1**) for all species with a range from 0.79 mg/mm³ (*Alyssum serpyllifolium* in CS) to 1.71 mg/mm³ in *Fumana thymifolia* (CS)).

**Table 1:** Functional trait values for all species and shrubland types weighted values.

Traits	Overall		HS shrubland		CS shrubland	
	Mean ± SD	CV (%)	Mean ± SD	CV (%)	Mean ± SD	CV (%)
**H**	40.73 ± 30.14	74.01	55.36 ± 34.69	62.66	34.54 ± 22.76	65.89
**SLA**	8.43 ± 5.11	60.60	7.76 ± 3.89	50.13	9.25 ± 6.32	68.32
**LA**	123.27 ± 299.96	243.33	126 ± 259.59	206.02	136.99 ± 368.00	268.63
**SSD**	0.67 ± 0.17	25.48	0.68 ± 0.15	22.06	0.67 ± 0.18	26.87

Mean value and standard deviation (Mean ± SD), Coefficient of Variation (CV, defined as SD/Mean). H: plant height (cm), SLA: specific leaf area (mm^2^/mg), LA: leaf area (mm^2^), and SSD: stem specific density (mg/mm³). Serpentine Mediterranean shrubland communities: CS and HS. The trait values for each individual species are presented in **Supplementary Material 2 Table S1**.

**Figure 1:**
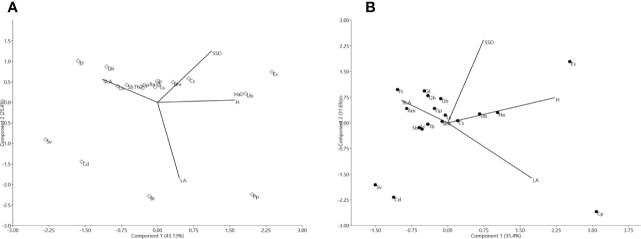
**(A)**. Biplot spatial diagram for the taxa of the *Halimium* shrubland (HS). The solid black dots are the studied taxa, identified with abbreviations indicated in **Supplementary Material 2 Table S1**. Traits are represented using abbreviations as follows: H: plant height (cm), SLA: specific leaf area (mm^2^/mg), LA: leaf area (mm^2^), and SSD: stem specific density (mg/mm³). **(B)**. Biplot spatial diagram of the taxa of the *Cistus* shrubland (CS). Solid black dots are the taxa, identified with abbreviations indicated in **Supplementary Material 2 Table S1**. Traits are represented using abbreviations as follows: H: plant height (cm), SLA: specific leaf area (mm^2^/mg), LA: leaf area (mm^2^), and SSD: stem specific density (mg/mm³).

The leaf traits presented a coefficient of variation between 20% and 60% for all species except for LA (**Table 1**). Although mean values for H and SSD were slightly higher in HS than in CS (**Table 1**), the ANOVA revealed significant differences for H between the two shrublands studied (F= 4.36; P<0.05). See **Supplementary Material 2 Table S1**. For the HS shrubland, the first two PCA axes explained 71.5% of the variance (**Figure 1A**; see **Supplementary Material 4 Table S1**). The first reflects a gradient of H representing *Halimium atriplicifolium*, *Phlomis purpurea*, *Cistus salvifolius*, *Ulex borgiae*, or *Erica scoparia* (SLA in the negative), while the second component is related to LA in the negative direction, represents species such as *Phlomis purpurea* and *Bupleurum rigidum* and has SSD in the positive direction. By comparison, for the CS shrubland, the first two PCA axes explained 67.08% of the variance (**Figure 1B**; see **Supplementary Material 4 Table S1**). The first axis reflects a gradient of H, whereas component 2 is related to SSD. In the positive direction for the first component are the tallest species, including *Halimium atriplicifolium*, *Cistus salvifolius* or *Erica scoparia* and, *Cistus populifolius* with a high LA, although *Ulex borgiae* is taller but had no leaves (LA non-existent). In the positive direction for the second component are species with a high SSD such as *Alyssum serpyllifolium* subsp. *malacitanum*, *Genista hirsuta*, *Genista triacanthos* or *Fumana thymifolia*.

We registered the species trait characteristics to the online TRY database (https://www.try-db.org/TryWeb/Home.php).

### 3.2 Functional group trait richness of the Mediterranean Iberian serpentine ecosystems

#### 3.2.1 Functional richness of the *Halimium* shrubland

Our cluster analysis revealed eight primary FGs coexisting in the HS (correlation coefficient 0.98; see **Supplementary Material 4 Figure S1**). The occurrence of eight FGs among 18 species corresponded to a SRi value of 0.44.

**Figure 2:**
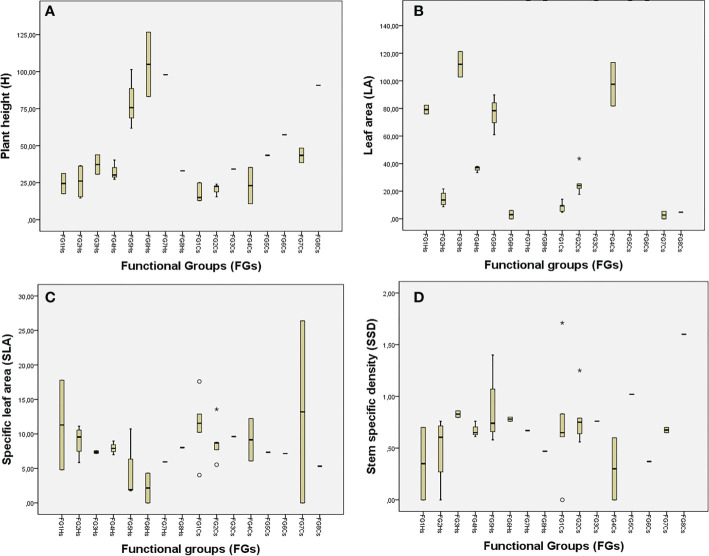
Mean trait values for: **(A)** H (plant height, cm), **(B)** LA (leaf area, mm^2^), **(C)** SLA (specific leaf area, mm^2^/mg), and **(D)** SSD (stem specific density, mg/mm³). The traits are grouped into the obtained functional groups at each shrubland. *Halimium* shrubland (HS) and *Cistus* shrubland (CS).

At the first step, species were clustered into two general groups: species with high LA or reduced LA, thereby indicating that the LA is a key trait. The FGs were primarily based on the mean values of FTs **Figure 2**; see **Supplementary Material 5 Table S1**). Values for FTs in each FG are expressed as the average of the component plants as follows:

- FG1 consists of small chamaephytes of reduced H (24.38 cm) and high LA (79.18 mm^2^) but reduced SSD. Those plants present the biggest SLA (17.79 mm^2^/mg), for instance, *Euphorbia flavicoma*.

- FG2 consists of small chamaephytes of reduced H (an average of 25.30 cm), reduced LA (14.4 mm^2^), and reduced SSD (0.48 mg/mm³). Its species include plants such as the obligate serpentine *Genista hirsuta* subsp. *lanuginosa*, that present stem-like leaves for most of the year.

- FG3 consists of small chamaephytes of greater H than plants in FG2 (37.24 cm) but with higher LA (112.02 mm^2^, highest after FG7 and FG8), and with higher values of SSD (0.78 mg/mm³). This group includes *Brachypodium retusum* and *Bupleurum acutifolium*.

- FF4 consists of small chamaephytes with lower H, LA, and SSD than among plants in FG3 (32.60 cm, 36.23 mm^2^ and 0.62 mg/mm³, respectively), but with higher SLA (7.95 mm^2^/mg), including e.g. *Staehelina baetica*.

- FG5 consists of nanophanerophytes with trait values in SSD and SLA similar to FG 3´s (0.69 vs 0.62 mm^2^/mg and 7.49 vs 7.95 mm^2^/mg), but with a greater H (79.61 vs 32.60 cm) and higher LA (76.38 vs 112.02 mm^2^). Such plants are situated just at the limit between nanophanerophytes (e.g., *Halimium atriplicifolium*) and chamaephytes (e.g., *Lavandula stoechas*) according to Raunkiaer (1934).

- FG6 and FG7 are both composed of nanophanerophytes with H values around 100 cm. However, whereas FG6 includes some spinescent plants with stem-like leaves (such as *Ulex borgiae*) or leaves with a reduced LA (such as *Erica scoparia*, with the smallest LA in the HS, at 6.02 mm^2^), FG7 includes plants with the biggest LA (758.78 mm^2^, including e.g. *Phlomis purpurea*). SSD was similar in the species between the two groups (0.67–0.75 mm^2^/mg).

- FG8 contained a only *Bupleurum rigidum*, which exhibits trait values similar to FG3´s, but is the chamaephyte that presents the maximum values of LA (670.07 mm^2^). It also stands out for its low value of SSD (0.47 mm^2^/mg), the lowest after FG1.

#### 3.2.2 Functional richness of the *Cistus* shrubland

We also identified eight FGs in the CS (correlation coefficient 0.99; functional groups are shown in see **Supplementary Material 4 Figure S2**). The occurrence of eight FGs in 19 species correspondes to a SRi value of 0.42.

As with the *Halimium* shrubland, LA was a key trait that distinguishes two groups at the first step. FGs were further derived based on the rest of the traits as follows (**Figure 2**; **Supplementary Material 5 Table S1**):

- FG1 consists of small chamaephytes with the lowest H values (18.12 cm) and the smallest LA (8.67 mm^2^), albeit without taking into consideration plants with ephemeral leaves or no leaves whatsoever. Species in this group have the second-highest values for SLA (11.30 mm^2^/mg) after plants in FG7. *Fumana thymifolia* stands out with a high SSD and SLA.

- FG2 consists of small chamaephytes with higher H, LA, and SSD values than species in FG1 (20.68 vs 18.1 cm; 20.54 vs 8.67 mm^2^ and 0.68 vs 0.54 mg/mm³, respectively), but smaller values of SLA (8.85 vs 11.25 mm^2^). *Alyssum serpyllifolium* stands out with a high LA, and *Galium boissieranum* with a high SSD.

- FG3 and FG6 consist of *Cistus* species that are separated primarily due to the reduced SSD present in *C. populifolius* compared with *C. salvifolius*, and because *C. populifolius* was the species with the highest LA value (1555.56 mm^2^) of all species.

- FG4 consists of *distachya* and *Lavandula stoechas*, small chamaephytes of H similar to plants in FG3, but with LA values (97.57 mm^2^) between those obtained for plants in FG3 and FG5.

- FG5 consists of the nanophanerophyte *Halimium atriplicifolium*, which has a high H value (43.52 cm), high LA (318.26 mm^2^ highest after FG6), and high SSD (0.74 mg/mm³; highest after *Erica*).

- FG7 and FG8: these FGs can be considered as nanophanerophytes due to a dwarfism syndrome found in *Ulex borgiae* and *Genista*, both of which are normally taller. Both groups include spinescent plants with stem-like leaves instead of leaves (e.g., *Ulex borgiae*), or with leaves showing the lowest LA (e.g., *Genista triacanthos*, LA: 6.02 mm^2^ and *Erica*, LA: 4.79 mm^2^). FG8 has higher values of SSD than plants in FG7 (0.9–0.64 mm^2^/mg respectively).

### 3.3 Dominant drought avoidance strategies and relationship to serpentine affinity

**Figure 3:**
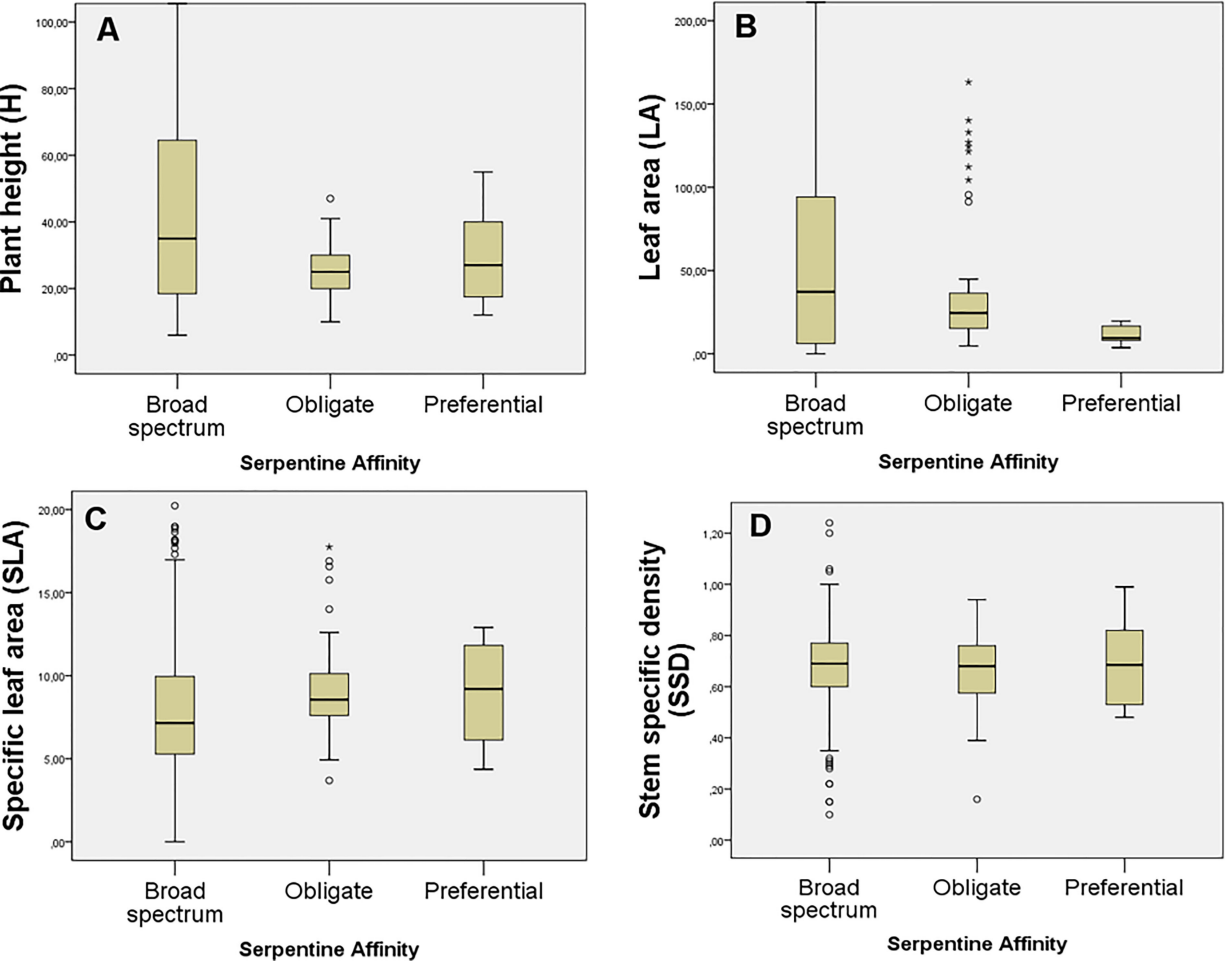
Mean trait values for: **(A)** H (plant height, cm), **(B)** LA (leaf area, mm^2^), **(C)** SLA (specific leaf area, mm^2^/mg), and **(D)** SSD (stem specific density, mg/mm³). The traits are grouped into the three serpentine affinity groups (O: Obligate; P: Preferential; and BS: broad-spectrum).

Although the general mean values for H, SLA, LA, and SSD were higher among the lower-affinity categories (i.e., P and BS; **Figure 3**), there were no significant differences between the FTs among the serpentine affinity of the studied plants (p> 0.05). The value trend for H increased such that BS > P > O (**Figure 3A**), for SLA increased such that P > O > BS, meaning that obligate serpentine species showed a slightly higher SLA (**Figure 3C**), and for LA increased such that Bs>O>P (**Figure 3B**). No value trend was detected for SSD (**Figure 3D**).

**Figure 4:**
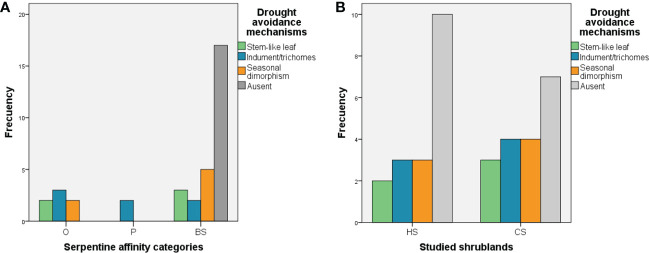
**(A)** Frequency of drought-avoidance mechanisms according to Travlos and Chachalis (2008) between the serpentine affinity categories. **(B)** Frequency of drought-avoidance mechanisms according to Travlos and Chachalis (2008) between the two serpentine types of shrublands. *Halimium* shrubland (HS) and *Cistus* shrubland (CS).

The number of species exhibiting drought avoidance mechanisms varied significantly between the serpentine affinity categories (*X* ^2^ = 18.54, df = 6, p = 0.005; **Figure 4A**; see **Supplementary Material 2 Table S1**). Obligate serpentine plants showed a high presence of trichomes, followed by seasonal dimorphism and stem-like leaves, while the majority of generalist species did not show drought avoidance mechanisms at all. However, the number of species with drought avoidance mechanisms did not vary significantly between the two types of serpentine shrublands, which had similar frequencies of such mechanisms (**Figure 4B**; see **Supplementary Material 2 Table S1**).

## 4 Discussion

### 4.1 Variation and trends in plant FTs

The reduced LA, H, SLA, and SSD documented in this region are in accordance with the leaf economics spectrum of other serpentine ecosystems around the world (Anacker et al., 2011; Harrison and Rajakaruna, 2011; Fernandez-Going et al., 2012). All measured FTs varied across the species studied. With respect to H, it was lower in obligate serpentine species than in species with lower serpentine affinity. The minimum and maximum values for H were recorded by generalist species such as *Erica scoparia*, which has a high affinity for European heathlands (Ojeda et al., 1996). The average height of the studied obligate and preferential serpentine plants in this region was 23 cm, which is far lower than that reached by *E. scoparia* (126.64 cm in CS) in another studied region (Ojeda et al., 1996). The reduced H detected in most of the studied species, especially for obligate serpentine plants, leads us to classify those plants primarily among small chamaephytes. In general, a comparison of plant heights in relation to the same plants inhabiting other, non-serpentine soils (Blanca et al., 2011), reveals that their height is reduced on serpentine soils (Sudarmono, 2007). Those generalists have modified their FTs to adapt to the serpentine ecosystem with an apparent specialization, probably due to the process of serpentinomorphoses (Pichi-Sermolli, 1948; Hidalgo-Triana and Pérez Latorre, 2019). H was the only FT that showed significant differences between our two shrubland types, with higher values in the HS shrubland. The shorter heights in CS could be explained by its higher altitude, where cold and wind can limit the length of the growing season (Körner et al., 1989). By contrast, the taller plants of the low-elevation HS intercept more light, though the trade-off may be that construction and maintenance costs and risk of breakage are increased (Poorter et al., 2008).

Another result that confirms a general adaptation of plants to Mediterranean climate and serpentine soils is reduced SLA, which provides a good leaf economic balance (Anacker et al., 2011). Our study, similar to other studies on serpentine ecosystems (Fernandez-Going et al., 2012; Adamidis et al., 2014), revealed that plants in serpentine ecosystems generally have low SLA. Reduced SLA is associated with stress tolerance and promotes nutrient retention, the reduction of water loss, and lowered susceptibility to desiccation (Navarro and Hidalgo-Triana, 2021). Compared with SLA of plants in other Mediterranean shrubland ecosystems, including coastal dunes in southern Spain (Rodríguez-Gallego et al., 2015), Mediterranean shrublands in Andalusia (Navarro and Hidalgo-Triana, 2021) and Iberian *Cistus* shrublands (Pérez Latorre and Cabezudo, 2002), the values for SLA detected in our study were small. Although Harrison et al. (2017) have demonstrated that serpentine plants have a lower SLA than non-serpentine plants, we did not find a significant effect of the serpentine affinity category, perhaps because we sampled plants inhabiting serpentine ecosystems with different degrees of affinity, whereas Harrison et al. (2017) studied both serpentine and non-serpentine ecosystems. Even so, the trend in SLA that we found (P>O>BS) demonstrates that SLA in obligate serpentine plants is slightly higher than in the other serpentine affinity categories. Generalists’ species growing in those serpentine soils also showed relatively lower SLA. However, because there is currently little information on the FTs of generalist species in serpentine soils, our database is the first available for serpentine plants on the Iberian Peninsula. For example, the same species *Lavandula stoechas*, a generalist species that can live in serpentine ecosystems, has been studied in non-serpentine ecosystems such as dunes and *Cistus* shrublands, among others (TRY; Kattge et al., 2011). The fact that this species has shown a higher SLA outside of serpentine ecosystems than in our study lends credence to Harrison et al. (2017) ´s idea that species that invest more resources in structural compounds and thus present higher values of leaf dry matter content -LDMC- (low SLA values) and thicker leaves (i.e., denser leaves) are more conservative (Zanzottera et al., 2020), as shown in serpentine ecosystems (Ackerly et al., 2002). At the same time, exploitative species tend to acquire resources rapidly and present high values of SLA (e.g. low values of LDMC) along with high relative growth and photosynthesis rates, which point to differences in functional strategies followed by the same species in different stressful environments (Zanzottera et al., 2020).

Leaf size is a FT in relation to architectural constraints and energy balance (soil nutrients, light, water, and temperature; Wright et al., 2017). In our work, although we did not detect significant statistical differences in LA depending on serpentine affinity, while LA of serpentine plants (i.e., obligate and preferential serpentine plants) was clearly lower than with the generalists, similar to what we detected for H. The mean value was an order of magnitude lower in the case of obligate serpentine plants. It is well known that leaf size, or LA, is smaller in dry, nutrient-poor environments such as serpentine habitats (Ackerly et al., 2002; Adamidis et al., 2014). We extend that insight by adding that generalist species in serpentine ecosystems have adapted their FT by reducing their leaf size in such a harsh environment.

By some contrast, SSD is a FT related to stem economics in terms of water conditions (Chave et al., 2009) and growth rate (Cornelissen et al., 2003). Plants with low SSD show a high rate of growth and high wood density that correlate with resistance to drought stress (Hacke et al., 2001). The average SSD value obtained in our serpentine ecosystems was higher (0.67 mg/mm³) than for other Mediterranean plants (Castro Díez and Montserrat Martí, 1998), and without differences among the three serpentine affinity categories. Among the categories indicated by Castro Díez and Montserrat Martí (1998), our plants are considered to be semi-woody plants, with hardwood at the base (SSD ≥0.6 mg/mm³) or soft, woody bases (<0.6 mg/mm³). Our higher values could be explained by the lower growth rate of our serpentine species and by the fact that evergreens have denser stems than deciduous shrub species.

We found that, in most cases, the larger the H, the higher the SSD, as others have similarly detected in other types of ecosystems (Shah et al., 2019). However, *Galium boissieranum*, a very small plant, had a high SSD, and a similar effect was found in the chamaephyte *F. thymifolia*, which showed SSD similar to a phanerophyte. The serpentine obligate *G. hirsuta* subsp. *lanuginosa*, which presented reduced H, also stood out for its high SSD, which could be explained by the intertwining of its branches. In general, the stem provides the structural strength that a plant needs to stand upright and the durability that it needs to live sufficiently long (Cornelissen et al., 2003). SSD plays an important role in a trade-off between the (relative) plant growth rate (which is high rate at low stem densities; Niklas, 1993) and stem defences against pathogens, herbivores and/or physical damage by abiotic factors (e.g., higher defence with higher stem densities). In combination with traits related to plant size, SSD also plays an important role in above-ground storage of carbon (Cornelissen et al., 2003). The persistence, stiffness, and longevity of stems could explain the differences obtained in SSD for the same species but in different communities (Castro Díez and Montserrat Martí, 1998). We recorded SSD being zero if a species had no recognizable above-ground support structure (Cornelissen et al., 2003) such as *Carex distachya* and *Sanguisorba verrucosa*, which are not obligate serpentine species.

Our principal component analysis revealed that the ecological strategies of coexisting plant species in both serpentine communities can be reduced to a pair of integrated dimensions, with H (and SLA in the negative) in one dimension, and a combination of LA and SSD in the other. Díaz et al. (2016) has identified two independent global dimensions of FT variation related to plant size and construction costs for photosynthetic LA, which suggests high levels of integration among FT. We demonstrated the same level of integration for the H and SLA of serpentine plants of the Southern Iberian Peninsula.

These indicator traits from those two dimensions (i.e., plant height, SLA, leaf size, and SSD) explained 67–72% of the variability in the trait matrix for our two communities. Those four traits alone may be sufficient for efficiently and accurately characterizing the strategies of coexisting species in trait-based models (De Bello, 2011; Lavorel et al., 2010). However, it would be interesting to study other FTs, such as diaspora mass, to describe the balance between seedling competitiveness and survival on the one hand, and dispersal and colonization ability on the other, along with nutrient-related traits such as Westoby (1998) defined leaf-height-seed plant ecology strategy framework, which has been supported by others (Laughlin et al., 2010; Díaz et al., 2016).

Both the LA and SLA of our shrubland species were reduced compared with average trait values for the same species found in other Mediterranean ecosystems. However, Ackerly et al. (2002) has shown that LA and SLA were not significantly correlated across species, which of our principal component analysis corroborates, thereby suggesting that those two traits are decoupled and associated with different aspects of performance along the peridotite-affinity gradient.

### 4.2 Functional groups of the Mediterranean Iberian serpentine ecosystems

Our results are consistent with the idea that dry and infertile serpentine soils are dominated by a mixture of FG (Hobbie et al., 2012; Hidalgo-Triana et al., 2018) and that adaptation strategies in serpentine ecosystems are very diverse. Our high number of FGs (functional group richness, FGR), could be used as an approximation of the functional diversity of an ecosystem (Wright I. J. et al., 2006), while our results concerning FGs for different FTs allow identifying the primary adaptations to ultramafic stress by shrublands. However, despite the phytosociological differences between the two communities, our results indicate that serpentine shrublands indeed share ecological strategies.

We obtained plant functional groups applicable to the shrublands types of Sierra Bermeja, and extensible to the rest of serpentine areas of Spain, according to functional adaptive attributes which can be used to manage ecosystems (Díaz and Cabido, 1997). There are few studies using cluster analysis on functional traits (e.g. García-Mora et al., 1999; Rudner and Groß, 2012), and studies for shrubland serpentine ecosystems are particularly lacking. Our finding of eight plant functional groups in two serpentine shrubland types that contain only 18 or 19 species, shows that there is a high level of functional diversity, even though some of FG classes are rare, containing only one species that does not share traits with the rest of the species. These species may deserve more attention.

In relation to other studies in different Mediterranean ecosystems, we identified the same number of FGs but with far fewer species. For example, Navarro et al. (2010) found six groups among 84 plant species in the Moroccan High Atlas Mountains, while and Grace et al. (2007) identified 11 FGs (in a total of 57 species) in Californian serpentine communities using environmental attributes such as climate and geology. Based on our results and those obtained in other studies on serpentine ecosystems, we can confirm that serpentine ecosystems could be highly resistant to environmental disturbances. For these reasons, our results are useful contextual information for understanding plant response to potential increases in drought caused by global warming (Zait and Schwartz, 2018).

### 4.3 Effect of the different serpentine affinities in shrubland communities and their drought avoidance mechanisms

Although the value trend for H increased such that BS > P > O, and for SLA increased in reverse order, we did not detect significant differences between the FTs among the serpentine affinity categories of the species studied. That fact indicates that generalist and obligate serpentine plants species inhabiting those serpentine ecosystems are affected by the peridotite rock independent of the level of serpentine affinity. Given the divergent phylogenetic histories of serpentine and generalist plants, it comes as no surprise that we found variation and different trends among the serpentine generalist and obligate serpentine taxa studied and in the relative habitat divergence that accompanies serpentine adaptation (Sianta and Kay, 2021).

However, we did not observe any clear pattern of community-level trait variation reflecting the plant economics continuum from acquisitive and fast-growing characteristics in pioneer succession stages to conservative and stress-tolerant features toward the succession climax. That could be because both serpentine shrublands we studied differ only in their floristic composition (in a phytosociological sense) and in elevation (Mota et al., 2017). Our studied shrublands share 13 species, of which eight are serpentine obligates with similar adaptive patterns. The lack of a strong environmental gradient in our study could explain the lack of community-level trait variation, but our study does cover the limited extent of serpentine shrub ecosystems in the Iberian Peninsula, which are found only distributed in Thermo and Mesomediterranean bioclimatic belts (Rivas-Martínez et al., 1996-2000).

With respect to drought avoidance mechanisms, our results confirm the existence of strong generalized responses to drought in Mediterranean climates, as has been shown for other Mediterranean areas (Sardans and Peñuelas, 2013; Garssen et al., 2014; Puglielli and Varone, 2018). However, we detected variation in drought avoidance mechanisms across the serpentine affinity categories, which confirms that the species with higher serpentine affinity can have higher drought tolerance. Our results agree with the results obtained by Harrison et al. (2017) in serpentine ecosystems in California and support Grime’s hypothesis, that linked soil infertility and stress-tolerant FTs confer unusually high resistance of plant species and communities to climate change.

Regarding drought avoidance mechanisms, we detected a relatively high content of trichomes in obligate serpentinophytes in contrast to glabrescence syndrome observed by Pichi-Sermolli (1948) and Hidalgo-Triana and Pérez Latorre (2019). An important role of the indumentum covering the leaf surface is the absorption of harmful UV-B radiation (Agati et al., 2012). As Harrison et al. (2017) has suggested for serpentine herbs, the shrublands in our study are subject to a very stressful habitat (Rostami et al., 2021), due to poor shading, particularly in the HS, which is located at low elevation that coincides with a reduced capacity for moisture retention, as pointed out by Rajakaruna and Boyd (2014).

The general presence of seasonal dimorphism detected by other authors in similar Mediterranean ecosystems (e.g. Orshan, 1964; Puglielli and Varone, 2018) also occurs in those ecosystems, albeit with a stronger representation in generalist species than in serpentinophytes. Our results suggest that the strategy is more generalized to Mediterranean species than serpentine plants, consistent with observations that Mediterranean woody species have developed structural, morphological, and physiological leaf traits that allow them to survive summer drought stress (Harley et al., 1987), in our case, photosynthetic oscillation.

The fact that the number of species with drought-avoidance mechanisms did not vary significantly between the two serpentine shrublands reinforces our hypothesis that both serpentine shrublands are adapted to summer drought. However, the study of other features representing adaptations to drought including amphistomaticy, the presence of a multilayered palisade of two to four layers, the reduction of stomatal conductance during drought, and the development of a prolific root system, could be studied in the future to narrow the gaps revealed by our study related to water use efficiency (Colombo et al., 2007; Travlos and Chachalis, 2008).

However, we are also cautious in that respect, because the future survival of those endemic-rich communities in infertile soils could be undermined by other factors such as nutrient availability, plant competition, and habitat loss (Damschen et al., 2012). Beyond that, Harrison et al. (2015) has suggested that for herb communities on serpentine soils, the benefit of more stress-tolerant FTs is counterbalanced by the disadvantage of less shading.

## 5 Conclusions

Most obligate and generalist taxa inhabiting the serpentine shrublands on the Southern Iberian Peninsula show the same ecological strategies: reduced plant size in comparison to the same species in other Mediterranean ecosystems, reduced SLA (i.e. thicker and/or denser leaves), and high SSD, with hairy and small-sized leaves. However, the detection of extensive drought avoidance mechanisms in serpentine plants, compared with their generalist peers, highlights that serpentine plants are likely to be less sensitive to climate change than species in other Mediterranean ecosystems.

The high number of FGs obtained with eight as the optimum number of clusters, shows that the serpentine shrublands are composed of species with a wide ecological range of FTs. That high number of FGs may both inform and challenge the conservation management of the vegetation. For plants whose FTs indicate high resilience to climate change, the objective may become avoiding an increase in the frequency of disturbances such as wildfire (Harrison et al., 2017). When an entire FG corresponds to only one species, more research and focused conservation assessments are likely needed.

## Data availability statement

The original contributions presented in the study are included in the article/**Supplementary Materials**, further inquiries can be directed to the corresponding author/s.

## Author contributions

NH-T designed the experiment, conducted field and laboratory work, analyzed the data and wrote the manuscript; AP-L designed the experiment, contributed to field work and revised the manuscript. JT, AA and MR participated in interpretation of results and revision of the manuscript. All authors contributed to the article and approved the submitted version.
