# Supplementary material for: Adaptations to the stressful combination of serpentine soils and Mediterranean climate drive plant functional groups and trait richness
Source: Front Plant Sci. 2023 Mar 13;14:1040839. doi: 10.3389/fpls.2023.1040839 (PMC10040603; doi:10.3389/fpls.2023.1040839)
Supplement: Supplementary file 1 [file DataSheet_1.pdf]

Supplementary Material 1

Figure S1.- Location of the study sites in the southern Iberian Peninsula and in the ultramafic outcrop of Sierra Bermeja (Estepona, Malaga province, Spain) with the studied shrublands: *Halimium* and *Cistus* shrublands.

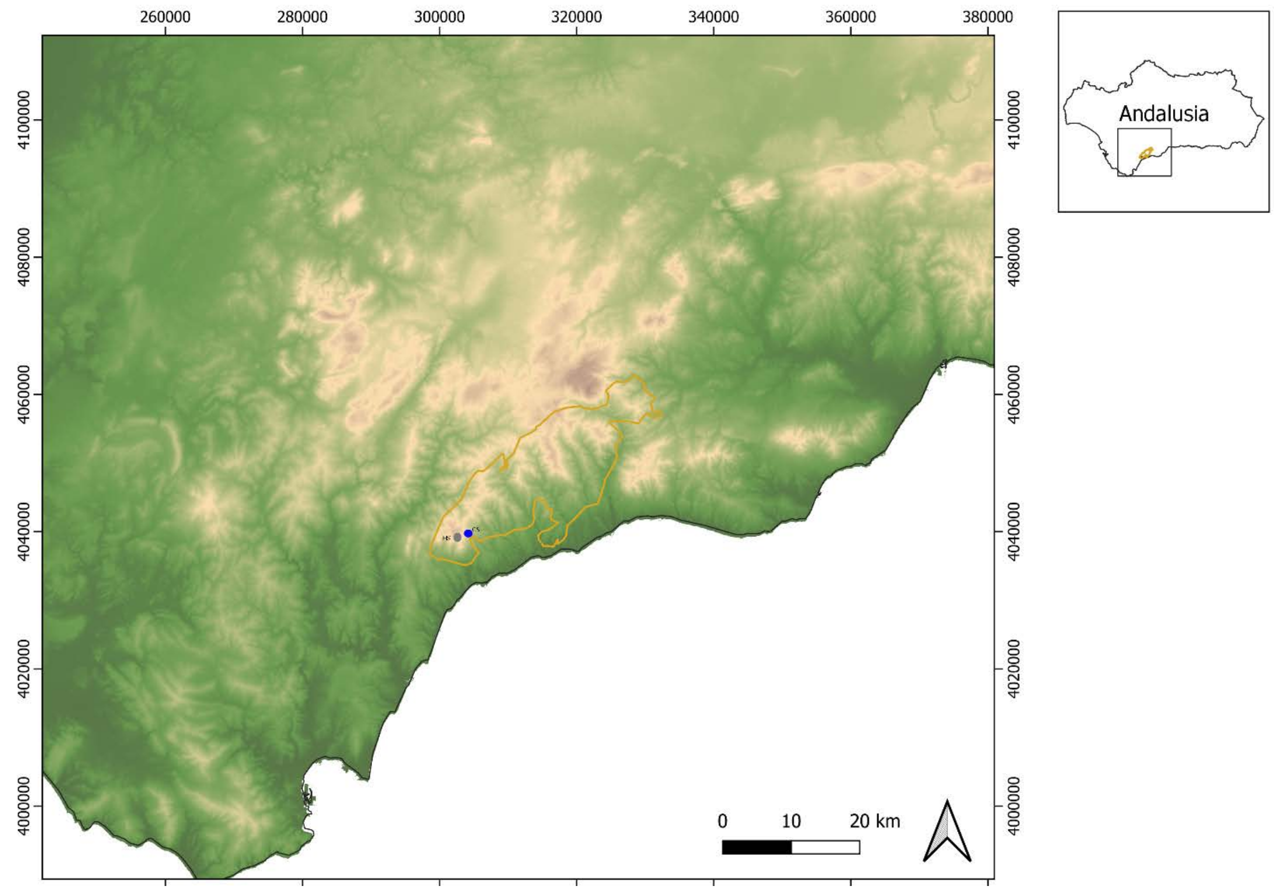

Supplementary Material 2

Table S1. Studied taxa. Serpentine affinity according to Pérez Latorre et al. (2013; 2018): O, Obligate; P, Preferential; Sub, Subserpentinophyte; BE, broad spectrum following Blanca et al. (2011). Scientific name and family name follow Blanca et al. (2011) and POWO (2019). HS: *Halimium* plant community; CS: *Cistus populifolius* shrubland**.** H: plant height (cm); SLA: specific leaf area (mm^2/mg); LA: leaf area (mm^2); SSD: stem specific density (mg/mm^3). Drought avoidance leaf traits follow Travlos and Chachalis (2008): seasonal dimorphism, Indument or trichomes, Stem-like leaf or absent (absence of drought-avoidance foliar trait).

ID	Taxa	Family	Code	Ultramafic plant community	Serpentine affinity	Mean Cover	H	SLA	LA	SSD	Drought avoidance mechanisms
1	*Bupleurum acutifolium* Boiss.	Apiaceae	Ba	HS	O	2	30.7	7.55	121.25	0.8	Seasonal dimorphism
2	*Brachypodium retusum* (Pers.) P.Beauv.	Poaceae	Bre	HS	BE	2	33,00	8.02	670.07	0.47	absent
3	*Bupleurum rigidum* L.	Apiaceae	Br	HS	BE	3	43.77	7.17	102.79	0.86	absent
4	*Carex distachya* Desf.	Cyperaceae	Cd	HS	BE	1	17.56	4.8	82.34	0.00	absent
5	*Cistus salvifolius* L.	Cistaceae	Cs	HS	BE	1	61.75	1.94	78.36	1.4	absent
6	*Erica scoparia* L.	Ericaceae	Es	HS	BE	2	126.64	4.3	6.02	0.8	absent
7	*Euphorbia flavicoma* DC.	Euphorbiaceae	Ef	HS	BE	2	31.2	17.79	76.01	0.7	absent
8	*Galium boissieranum* (Steud.) Ehrend. & Krendl	Rubiaceae	Gb	HS	P	1	36.48	5.85	15.32	0.67	Indument/trichomes
9	*Genista hirsuta* Vahl subsp. *lanuginosa* (Spach) Nyman var. *lanuginosa*	Fabaceae	Gh	HS	O	1	16.48	11.12	8.72	0.76	Stem-like leaf
10	*Glandora prostrata* (Loisel.) D.C.Thomas	Boraginaceae	Gp	HS	BE	1	40.28	7.9	33.45	0.65	absent
11	*Halimium atriplicifolium* (Lam.) Spach	Cistaceae	Ha	HS	BE	4	101.4	1.73	89.83	0.74	Seasonal dimorphism
12	*Lavandula stoechas* L.	Labiatae	Ls	HS	BE	1	75.68	10.73	60.94	0.58	absent
13	*Linum narbonense* L.	Linaceae	Ln	HS	BE	1	35.76	10.01	21.66	0.54	absent
14	*Phlomis purpurea* L.	Labiatae	Pp	HS	BE	2	97.96	5.94	758.78	0.67	Seasonal dimorphism
15	*Staehelina baetica* DC.	Compositae	Sb	HS	O	1	14.56	9.1	11.89	0.61	Indument/trichomes
16	*Sanguisorba verrucosa* (G. Don) Ces.	Rosaceae	Sv	HS	BE	2	30.2	8.97	37.18	0.61	absent
17	*Teucrium haenseleri* Boiss.	Labiatae	Th	HS	BE	1	27.32	6.98	38.07	0.76	Indument/trichomes
18	*Ulex borgiae* Rivas Mart.	Fabaceae	Ub	HS	BE	3	83.2	0.00	0.00	0.76	Stem-like leaf
						Mean ± SD	50.22±33.07	7.22±4.08	122.93±218.74	0.65±0.31	

19	*Alyssum serpillifolium* Desf. subsp. *malacitanum* Rivas Goday	Brassicaceae	Am	CS	O	1	15.44	13.56	22.15	0.79	Seasonal dimorphism
20	*Brachypodium retusum* (Pers.) P.Beauv.	Poaceae	Bre	CS	BE	2	23.96	5.53	43.62	0.64	absent
21	*Carex distachya* Desf.	Cyperaceae	Cd	CS	BE	1	10.75	6.08	113.35	0,00	Seasonal dimorphism
22	*Cistus salviifolius* L.	Cistaceae	Cs	CS	BE	4	57.36	7.15	1555.58	0.37	Seasonal dimorphism
23	*Cistus populifolius* L.	Cistaceae	Cp	CS	BE	1	34.2	9.62	169.62	0.76	absent
24	*Erica scoparia* L.	Ericaceae	Es	CS	BE	1	90.76	5.31	4.79	1.6	absent
25	*Fumana thymifolia* (L.) Webb	Cistaceae	Ft	CS	BE	2	12.96	17.6	9.42	1.71	absent
26	*Galium boissieranum* (Steud.) Ehrend. & Krendl	Rubiaceae	Gb	CS	P	1	22.36	7.71	17.64	1.25	Indument/trichomes
27	*Genista hirsuta* Vahl subsp. *lanuginosa* (Spach) Nyman var. *lanuginosa*	Fabaceae	Gh	CS	BE	2	25.12	12.87	5.43	0.83	Stem-like leaf
28	*Genista triacanthos* Brot.	Fabaceae	Gt	CS	BE	1	48.36	26.38	5.43	0.65	Stem-like leaf
29	*Glandora prostrata* (Loisel.) D.C.Thomas	Boraginaceae	Gp	CS	BE	1	22.84	8.68	23.99	0.75	absent
30	*Halimium atriplicifolium* (Lam.) Spach	Cistaceae	Ha	CS	BE	3	43.52	7.34	318.26	1.02	Seasonal dimorphism
31	*Linum carratracense* (Rivas Goday & Rivas Mart.) Mart.Labarga & Muñoz Garm.	Linaceae	Lc	CS	O	1	35.28	12.22	81.78	0.6	Indument/trichomes
32	*Lavandula stoechas* L.	Labiatae	Ls	CS	BE	1	24.76	10.24	9.61	0.61	absent
33	*Thymus baeticus* Boiss. ex Lacaita	Labiatae	Tb	CS	BE	1	12.77	11.54	14.15	0.65	Indument/trichomes
34	*Staehelina baetica* DC.	Compositae	Sb	CS	O	1	18.8	8.77	25.29	0.56	Indument/trichomes
35	*Sanguisorba verrucosa* (G. Don) Ces.	Rosaceae	Sv	CS	BE	2	15.00	4.02	4.75	0.65	absent
36	*Ulex borgiae* Rivas Mart.	Fabaceae	Ub	CS	BE	2	38.56	0.00	0.00	0.7	Stem-like leaf
						Mean ± SD	30.71±19.98	9.70±5.79	134.71±363.57	0.75±0.44	

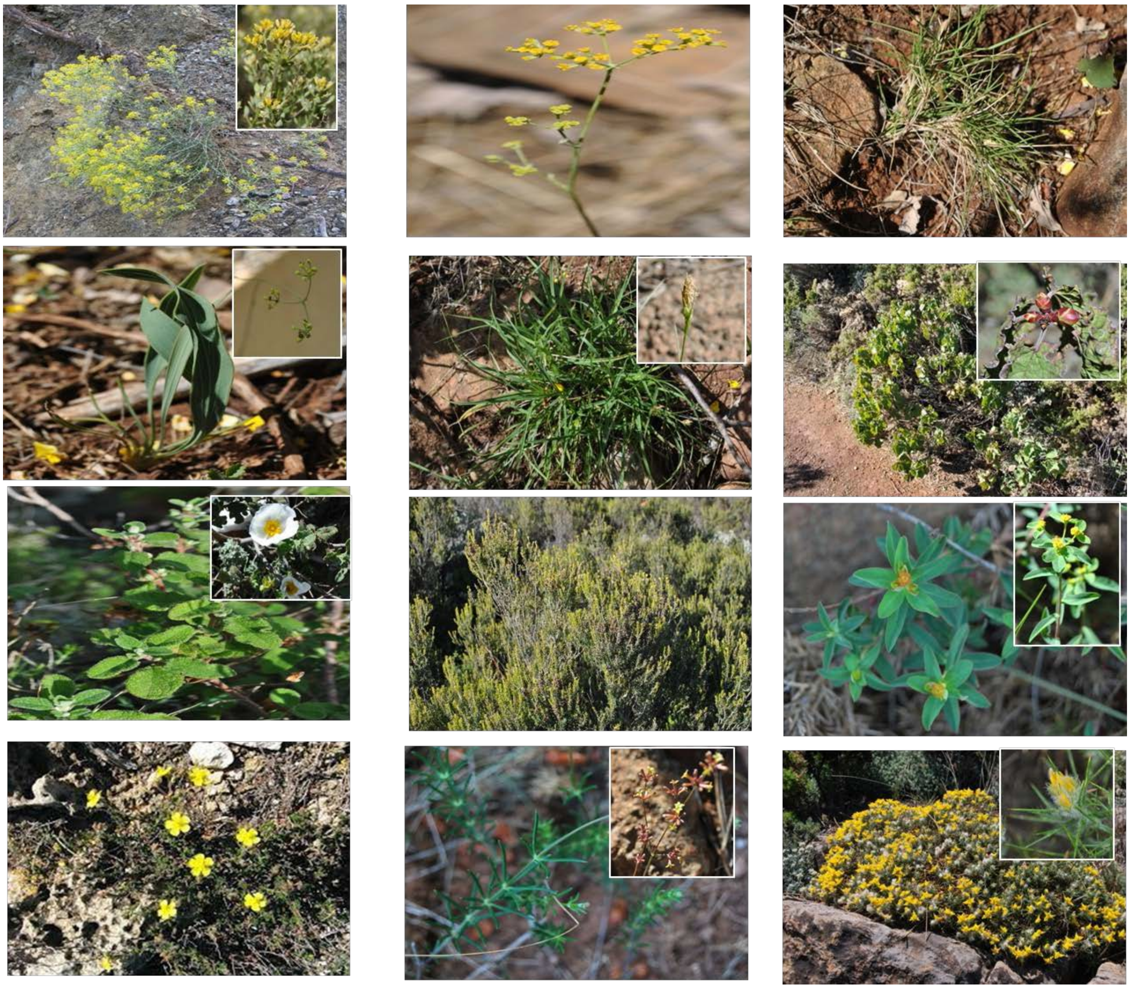

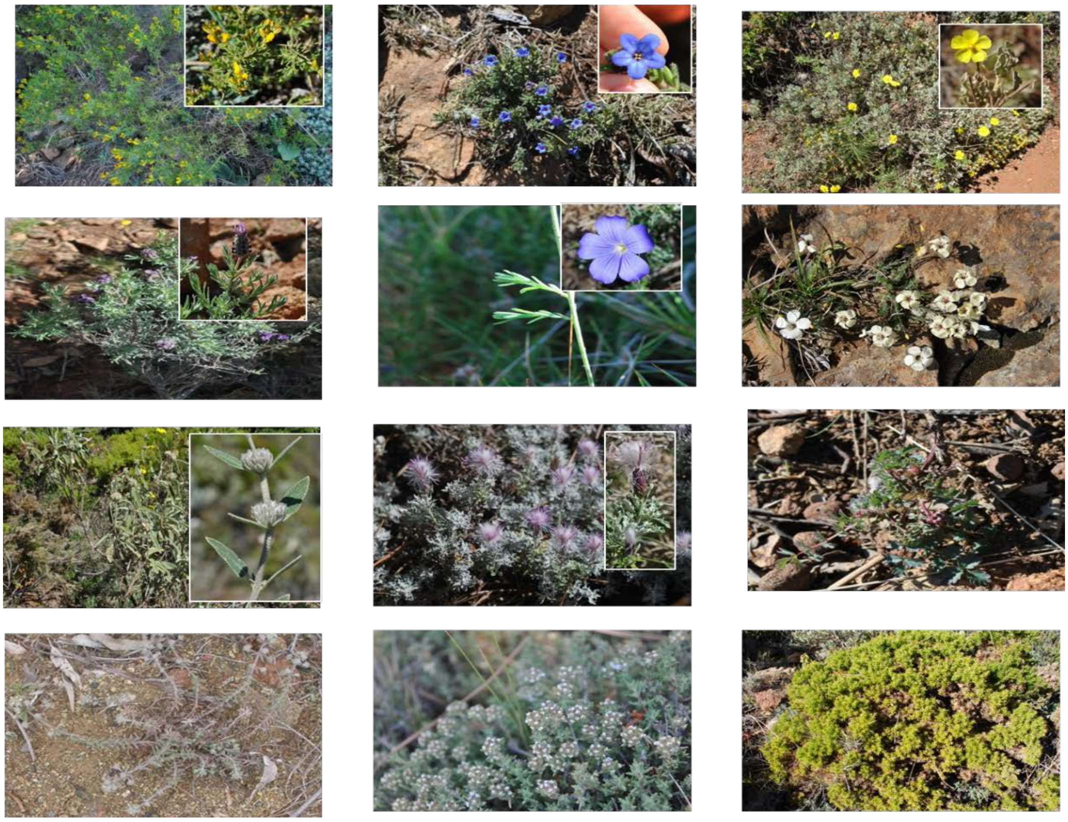

Figure S1. Photographs of the studies species. 1. *Alyssum serpyllifolium* subsp. *malacitanum*. 2. *Bupleurum acutifolium*. 3. *Brachypodium retusum*. 4. *Bupleurum rigidum.* 5. *Carex distachya*. 6. *Cistus populifolius*. 7. *Cistus salviifolius*. 8. *Erica scoparia*. 9. *Euphorbia flavicoma*. 10. *Fumana thymifolia*. 11. *Galium boissieranum*. 12. *Genista hirsuta* subsp. *lanuginosa* var. *lanuginosa.* 13. *Genista triacanthos* 14. *Glandora prostrata*. 15. *Halimium atriplicifolium*. 16. *Lavandula stoechas*. 17. *Linum narbonense*. 18. *Linum carratracense.* 19. *Phlomis purpurea.* 20. *Staehelina baetica. 21*. *Sanguisorba verrucosa.* 22. *Teucrium haenseleri.* 23. *Thymus baeticus.* 24. *Ulex borgiae*. Authors

Supplementary Material 3

Table S1. List of the plant functional traits assessed in the sampled species. For each trait we included: an abbreviation, units, protocol, the categories (for traits coded as binary or as ordinal variables); the associated ecological functions and the sample size according to Pérez-Harguindeguy et al. (2013).

**ID**	**Trait**	**Abbreviation**	**Sample size**	**Associated ecological functions**
1	Plant height	H	25	Response to disturbance and soil resources, competitive strength
2	Leaf area	LA	10	Energy balance, CO_2 and water exchange
2	Specific leaf area	SLA	10	Response to soil resources, plant defense
4	Stem specific density	SSD	10	Competitive strength

Supplementary Material 4

Supplementary Material 5 Table S1. Principal components analyses of leaf trait data of *Halimium* shrubland. Variables that contribute more at each component (PC) are indicated whit bold. Trait abbreviations: H-Height plant, LA- leaf area, SLA-specific leaf area and SSD-stem specific density.

	Halimium shrubland				Cistus *shrubland*			
	PC1	PC2	PC3	PC4	PC1	PC2	PC3	PC4
H	**0.69**	0.02	0.00	0.72	**0.73**	0.19	0.23	-0.61
LA	0.20	**-0.80**	0.54	-0.16	0.52	**-0.56**	0.31	0.56
SLA	-0.50	0.24	**0.69**	0.47	-0.32	0.23	**0.92**	0.04
SSD	0.49	**0.54**	0.48	-0.49	0.30	**0.77**	-0.11	0.55

Figure S1. Classification (dendrogram) of 18 plants of a serpentine community (*Halimium* shrubland) into 8 functional groups by functional traits similarity.

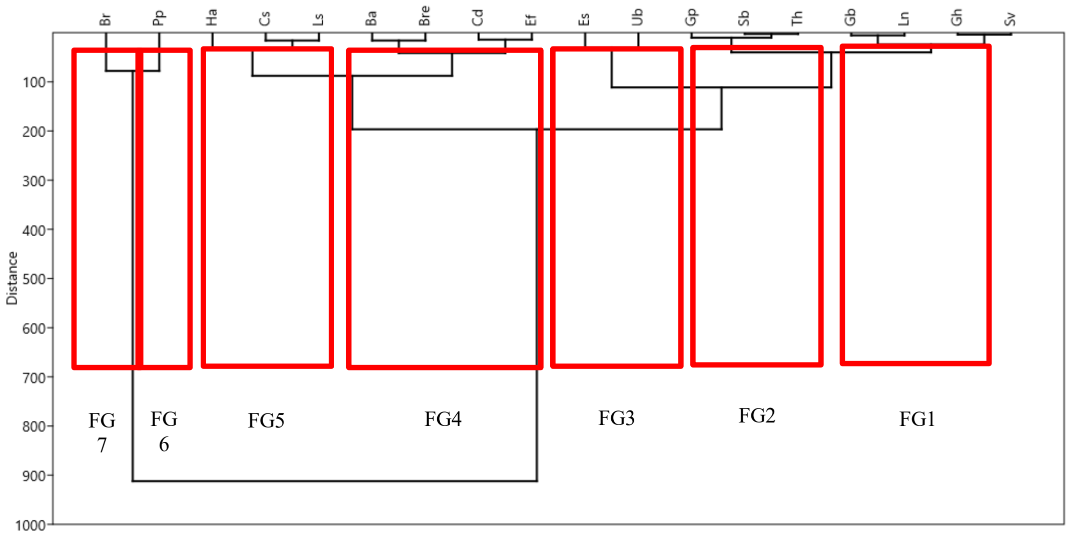

Figure S2. Classification (dendrogram) of 18 plants of a serpentine community (*Cistus* shrubland) into 8 functional groups by functional traits similarity.

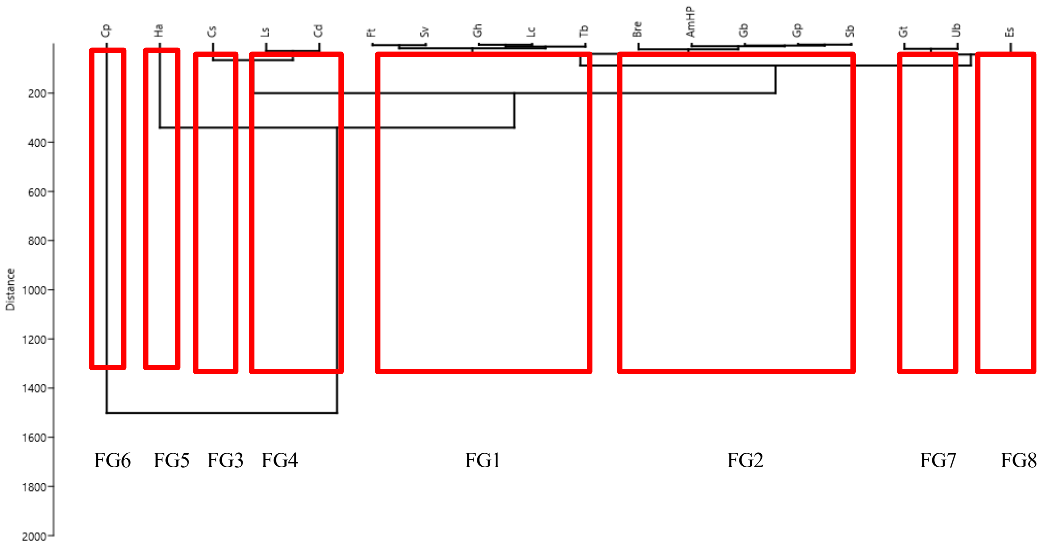

Supplementary Material 5

Table S1. FGs obtained for *Halimium* shrubland (HS) from cluster analysis. H: plant height (cm); SLA: specific leaf area (mm^2 /mg); LA: leaf area (mm); SSD: stem specific density (mg/mm^3). FG: functional group.

Scientific name/ Functional traits	Plant height	Leaf area	Specific leaf area	Stem specific density	Functional Groups
	**H**	**LA**	**SLA**	**SSD**	
C. distachya	17.56	82.34	4.80	0.00	**FG1:** Chamaephytes (small chamaephytes) of high LA but Reduced SSD (perennial herbs).
E. flavicoma	31.20	76.01	17.79	0.67	
G. hirsuta	16.48	8.72	11.12	0.70	**FG2:** Chamaephytes (small chamaephytes) of reduced LA but high SLA, reduced SSD (except Genista). Some of them with spinescence.
S. verrucosa	12.48	11.89	9.10	0.00	
G. boissieranum	36.48	15.32	5.85	0.67	
L. narbonense	35.76	21.66	10.01	0.54	
B. acutifolium	30.70	121.25	7.55	0.76	**FG3:** Chamaephytes of high LA but reduced SLA. High SSD and high H.
B. retusum	43.77	102.79	7.17	0.79	
G. prostrata	40.28	33.45	7.90	0.60	**FG4**: Chamaephytes of low H (small chamaephytes), reduced LA but SLA similar to FG3.
S. baetica	30.20	37.18	8.97	0.61	
T. haenseleri	27.32	38.07	6.98	0.65	
C. salviifolius	61.75	78.36	7.23	0.78	**FG5:** Chamaephytes-nanoPhanerophytes of high H and reduced SLA.
H. atripicifolium	101.40	89.83	4.23	0.71	
L. stoechas	75.68	60.94	10.73	0.58	
E. scoparia	126.64	6.02	4.30	0.80	**FG6:** Phanerophytes of reduced LA and SLA (*Ulex* is spinescent, without leaves).
U. borgiae	83.20	0.00	0.00	0.70	
P. purpurea	97.96	758.78	5.94	0.67	**FG7:** (nano) Phanerophytes, high LA and reduced SLA.
B. rigidum	33.00	670.07	8.02	0.47	**FG8**: Chamephytes of reduced SSD (perennial herb) but high LA.

Table S2. FGs obtained for the *Cistus* plant community (HS) from cluster analysis. H: plant height (cm); SLA: specific leaf area (mm^2/mg); LA: leaf area (mm^2); SSD: stem specific density (mg/mm^3). FG: functional group.

Scientific name/ Funtional traits	Plant height	Leaf area	Specific leaf area	Stem specific density	Functional Groups
	H	LA	SLA	SSD	
F. thymifolia	12.96	9.42	17.6	0.8	**FG1:** Chamaephytes and small chamaephytes with reduced LA, high SLA, and high SSD.
S. verrucosa	12.77	14.15	11.54	0	
G. hirsuta	25.12	5.43	12.87	0.76	
L. carratracense	24.76	9.61	10.24	0.51	
T. baeticus	15	4.75	4.02	0.65	
A. serpyllifolium	15.44	22.15	13.56	0.68	**FG2:** Chamaephytes and small chamaephytes with higher LA than FG1 but low SLA.
B. retusum	23.96	43.62	5.53	0.64	
G. boissieranum	22.36	17.64	7.71	0.8	
G. prostrata	22.84	23.99	8.68	0.71	
S. baetica	18.8	25.29	8.77	0.56	
C. salviifolius	34.2	169.62	9.62	0.62	**FG3:** Chamaephytes (small chamaephytes) with high LA.
C. distachya	10.75	113.35	6.08	0	**FG4:** Chamaephytes and small chamaephytes with medium LA and low SSD.
L. stoechas	35.28	81.78	12.22	0.6	
H. atriplicifolium	43.52	318.26	7.34	0.74	**FG5:** Nanophanerophyte with high H, high LA but SLA reduced. High SSD.
C. populifolius	57.36	1555.58	7.15	0.37	**FG6:** Nanophanerophyte with high H, high LA but SLA reduced. Low SSD.
U. borgiae	40.73	0	0	0.7	**FG7:** Nanophanerophytes with low SLA (spinescent shrubs, without leaves) and reduced H.
G. triacanthos	48.36	5.43	26.38	0.57	

E. scoparia	90.76	4.79	5.31	0.9	**FG8:** Nanophanerophyte with reduced LA but high SSD.
